# Short Practical Regimen of Acupuncture for Melasma: A Prospective Cohort Study in a Tertiary Hospital in Thailand

**DOI:** 10.3389/fpubh.2021.761017

**Published:** 2021-11-04

**Authors:** Thanan Supasiri, Nuntida Salakshna, Krit Pongpirul

**Affiliations:** ^1^Department of Preventive and Social Medicine, Faculty of Medicine, Chulalongkorn University, Bangkok, Thailand; ^2^Samitivej Esthetics Institute, Samitivej Sukhumvit Hospital, Bangkok, Thailand; ^3^Science Division, Mahidol University International College, Nakhon Pathom, Thailand; ^4^Department of International Health and Department of Health, Behavior, and Society, Johns Hopkins Bloomberg School of Public Health, Baltimore, MD, United States

**Keywords:** acupuncture–therapy, melasma, dose–response, melasma area and severity index, Thailand

## Abstract

**Background:** Acupuncture shows benefits for patients with melasma, although no optimal number of sessions have been determined.

**Methods:** The prospective observational study was conducted in melasma patients who were treated with acupuncture procedures two times a week and were evaluated after the 5th and the 10th sessions of acupuncture, with a 1-week follow-up after the last session. Participants Groups A and B received five and 10 acupuncture sessions, respectively. Melasma was assessed by using the melanin index (MI), melasma area and severity index (MASI), patient-reported improvement scores, and acupuncture-related adverse events.

**Results:** Out of 113 participants, 67 received five sessions of acupuncture treatment while 39 received 10 sessions. At 1 week after five sessions of acupuncture in Group A, the mean MI decreased by 28.7 (95% CI −38.5 to −18.8, *p* < 0.001), whereas the median MASI decreased by 3.4 (95% CI −6.9 to −1.2, *p* < 0.001) points. At 1 week after ten sessions of acupuncture in Group B, the mean MI decreased by 31.3 (95% CI −45 to −17.6, *p* < 0.001), whereas the median MASI decreased by 5.4 (95%CI −9.9 to −3, *p* < 0.001) points. The first five sessions of acupuncture had a higher incremental effect than the last five sessions, although there was no statistically significant difference. Twenty-nine participants reported minor side effects. Group B had a risk ratio (RR) of having adverse events 1.8 times (95% CI 1.0–3.4, *p* = 0.05) compared with Group A.

**Conclusion:** Short acupuncture regimens of 5–10 sessions in melasma seem to be effective and practical with minor side effects.

## Summary Box

What is already known about this subject?

- Acupuncture has effectively improved melasma conditions at low cost with minimal side effects.
- Traditionally, one acupuncture course comprises 10 sessions for a chronic condition but dose-response evidence of acupuncture for melasma has been lacking.
- The number of acupuncture sessions performed in various randomized controlled trials varied from 21 to 60 over 4–16 weeks.

What are the new findings?

- Five sessions of acupuncture improved the melanin index (MI) by 28.7 and melasma area and severity index (MASI) by 3.4 points.
- About 10 sessions of acupuncture improved MI by 31.3 and MASI by 5.4 points, of which the improvement was mainly from the first five sessions.
- Short acupuncture regimens of 5–10 sessions in melasma seem to be effective and practical with minor side effects.

How might it impact clinical practice in the foreseeable future?

- The dose-response study has not been common but could have clinical and financial implications.

## Introduction

Melasma is acquired hyperpigmentation characterized by ill-defined light to dark brownish macules and patches, typically affecting photo-exposed areas of the face (1–3). The most commonly implicated etiological factors are chronic ultraviolet exposure, female hormone stimulation, and genetic predisposition (4–6). Its appearance negatively impacts the quality of life, emotional wellbeing, and may even lead to psychiatric consequences (7, 8).

In the last decade, there was a paradigm shift in the pathophysiology of melasma which is now considered to be a more complex interplay between epidermal and dermal components including the proliferation of dermal vessels and oxidative damage by reactive oxygen species (ROS) (4, 5). Apart from conventional treatments such as hydroquinone, triple-combination cream (hydroquinone, tretinoin, and fluocinolone acetonide), and other bleaching agents, many new emerging therapies which target vascular components such as tranexamic acid or vascular targeting laser and light treatment (such as pulsed dye laser and intense pulsed light) have shown benefits in lightening the melasma area (9–12).

Acupuncture has shown benefits for patients with melasma. The effective rate of acupuncture for melasma has been reported to be around 87–96% (13–15). In Chinese medicine theory, acupuncture can promote blood and qi circulation, reducing blood stasis, rebalance and regulate various internal systems in the body to promote the metabolism of epidermal cells, as well as regulate several hormones and antioxidants thus improving the melasma (13, 16, 17). Another randomized control trial (RCT) found that after the acupuncture treatment for melasma, blood estrogen level decreased while progesterone increased, which corresponds to the association of increasing estrogen and decreasing progesterone level in melasma patients (14). Moreover, acupuncture for melasma has been shown to be effective at low cost with minimal side effects which is beneficial for this chronic recurrent skin disease. However, it requires multiple frequent treatments, which can be inconvenient for the patients. Traditionally, one course of acupuncture treatment for a chronic disease consists of 10 sessions (18), but the acupoints used and the optimal number of treatment sessions for acupuncture for melasma have been inconclusive. Therefore, this study aims to investigate and compare a short-fixed regimen of acupuncture between 5 and 10 sessions by body acupuncture with encircling and intralesional needling methods.

## Methods

### Study Design and Population

This prospective observational cohort was conducted at the Acupuncture Clinic, King Chulalongkorn Memorial Hospital, Bangkok, Thailand, from September–December 2018. We recruited all the melasma patients who came to the clinic for acupuncture treatment and were willing to participate in this observational study. We excluded participants who were pregnant, had severe underlying disease or psychosis, had a bleeding tendency, or had skin infections in the acupoints area. All participants were directed to maintain their usual behavior, such as using the same frequency and amount of sunscreen protection or continuing to not use sunscreen regularly.

### Acupuncture Procedure

**Figure 1:**
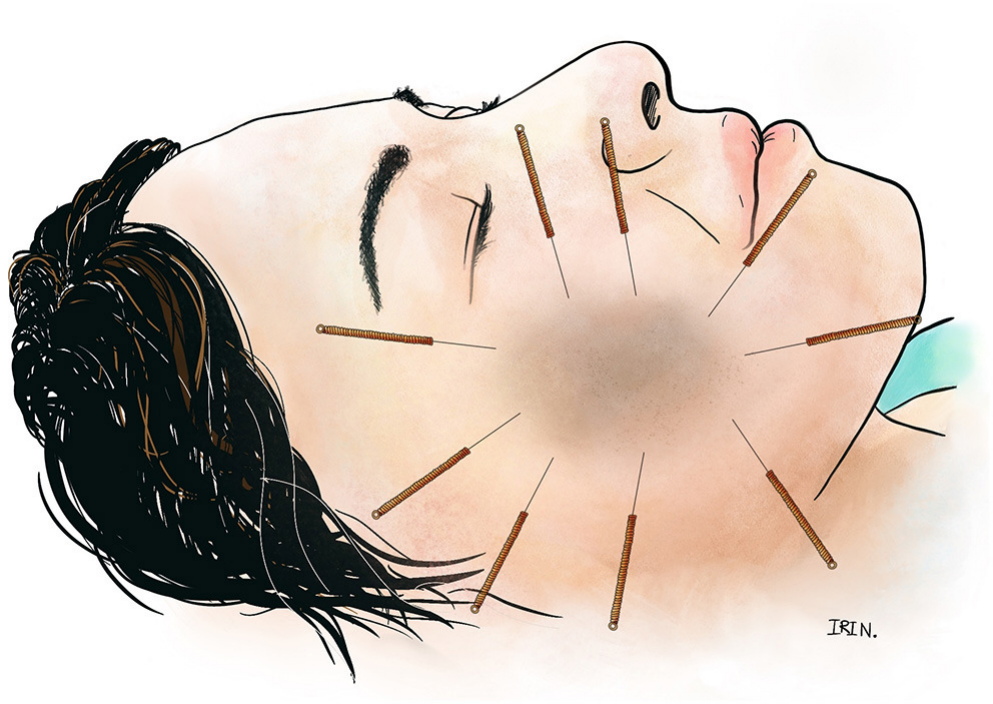
Encircle acupuncture of a facial melasma lesion.

**Figure 2:**
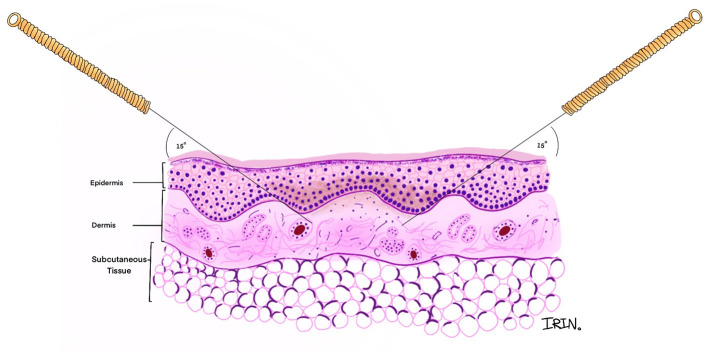
Cross-sectional view of angle and direction of the encircle acupuncture.

All the patients were treated with fixed acupoints: encircling the melasma area (Figures 1, 2), Fengchi (GB20), Hegu (LI4), Xuehai (SP10), Jusanli (ST36), Sanyinjiao (SP6), and Taichong (LR3). All acupoints were performed bilaterally except those encircling the melasma area. The acupoints were carefully selected from the literature review of standard teaching materials in China (18) and those frequently used in previous studies (13, 19). We used sterile disposable stainless steel needles sized.25 mm × 40 mm (EACU^tm^, Maanshan Bond Medical Instruments Co., Ltd., China). Acupuncture was done two times a week by well-trained acupuncturists, licensed with acupuncture Master degrees certified from China. The “De Qi” sensation in every acupoint was mandatory, and the needles were retained 30 min each time. Only manual needle-stimulation was used. The participants were evaluated at every fifth session of acupuncture. Since this was an observational study, we used the usual protocol used in our acupuncture clinic. If the first five sessions of acupuncture did not yield satisfactory results and the patients were willing to continue the treatment, we continued the acupuncture treatment for five more sessions. Therefore, participants were divided into two groups: Group A received five acupuncture sessions and Group B received ten acupuncture sessions.

### Outcome Measurements

The participants were evaluated at the first visit, every five sessions of acupuncture, and 1 week after their last session.

The primary outcome was the change in the melanin index (MI) measured by Mexameter® MX 18 (Courage+Khazaka electronic GmbH, Köln, Germany) on the melasma affected part of the face. The MI was calculated as the average of a triplicate measurement in the area where the melasma was darkest.

The secondary outcomes consisted of the change in the melasma area and severity index (MASI), patient-reported improvement scores, and related adverse events from acupuncture. The MASI was developed by Kimbrough-Green et al. (20) and assesses three factors—area (A) of involvement, darkness (D), and homogeneity (H)—at four locations: the forehead (f), right malar region (rm), left malar region (lm) and chin (c). The MASI score can be calculated using the following formula:

MASI score = 0.3A_f_(D_f_ + H_f_) + 0.3A_rm_(D_rm_ + H_rm_) + 0.3A_lm_(D_lm_ + H_lm_) + 0.1A_c_(D_c_ + H_c_)

The area of involvement is rated from 0 to 6 while the darkness and homogeneity of pigmentation are rated from 0 to 4; thus, the MASI score ranges from 0 (none) to 48 (severe melasma).

The subjective improvement scores were measured by the 6-point Likert Scale, from−1 to +4 (-1, worsening; 0, no improvement; +1, slight improvement; +2, moderate improvement; +3, marked improvement; +4, almost cured or completely cured). The adverse events due to acupuncture were recorded by interviewing the participants and physical examinations by physicians each visit.

All participants were also photographed at every evaluation visit. The MI, MASI, and satisfactory scores were evaluated by the same evaluator who has been trained by a dermatologist.

### Statistical Analysis

The baseline characteristics and data at each time point were analyzed by descriptive statistics. The data after treatment compared with the baseline in each group was analyzed by using a Student's paired *t*-test or Wilcoxon signed-rank tests as appropriate. Linear regression was used to compare the results between Group A and Group B after adjusting for unequal baselines. Linear regression was also used to see if any baseline factors are affecting the outcome. For Group B, a mixed model with the subject as a random effect was used to compare the result of the first five sessions (1st−5th) and the last five sessions (6th−10th). Pairwise comparison between the first and last five sessions was made using Scheffe's Test. If the data did not meet the assumptions to use the mixed model or linear regression, the nonparametric test was used. The data were analyzed using the Stata/MP 15.1 (StataCorp LLC, College Station, TX, USA) software package.

### Trial Registration

The study was registered on August 19, 2018, before recruiting the first case, with the Thai Clinical Trials Registry, Identification Number TCTR20180824002.

### Ethics Approval

The study was approved by the Institutional Review Board (IRB), Faculty of Medicine, Chulalongkorn University (IRB No.544/2559). Each participant provided written informed consent.

## Results

One hundred and thirteen melasma patients came for acupuncture treatment during September–December 2018. The baseline characteristics are shown in Table 1. The acupuncturists finished the course of treatment after five sessions for 67 participants due to the significant improvement achieved. The other 39 participants continued until 10 sessions. Seven participants did not complete at least five sessions of acupuncture and thus, were not evaluated.

**Table 1:** Characteristics of the participants.

	**Group A:**	**Group B:**
	**Five sessions of**	**Ten sessions of**
	**acupuncture treatment**	**acupuncture treatment**
	**(*n* = 67)**	**(*n* = 39)**
Female [*n* (%)]	64 (95.5)	37 (94.9)
Age [mean (SD)] (years)	44.3 (9.7)	52.5 (9.7)
Duration of melasma [mean (SD)] (years)	7.4 (6.8)	8.3 (6.2)
Prior acupuncture experience [*n* (%)]	18 (26.9)	13 (33.3)
Family history of melasma [*n* (%)]	48 (71.6)	22 (56.4)

### Melanin Index (MI)

The average baseline MI for all the participants in the darkest area was 298.9 (SD 79.7). Table 2 shows the baseline MI and measurements at each time-point for Groups A and B.

**Table 2:** Mean melanin index (MI) at each evaluation visit.

**Melanin index**	**Group A (*n* = 67)**	***p-*value**	**Group B (*n* = 39)**	***p*-value**
	**mean (SD)**		**mean (SD)**	
Baseline	287.4 (64.8)	Reference	315.9 (98.5)	Reference
After the 5th session	262.3 (62.5)	<0.001^*^	293.4 (90.6)	0.001^*^
One week after the 5th session	258.7 (61.4)	<0.001^*^		
After the 10th session			285.6 (94.7)	<0.001^*^
One week after the 10th session			284.6 (89.2)	<0.001^*^

* *p-value calculated using Student's paired t-test*.

In Group A, after the 5th session of acupuncture, the MI decreased by 25 (95% CI −34 to −16.1, *p* < 0.001): from 287.4 (SD 64.8) to 262.3 (SD 62.5). At 1 week after completing five sessions, the mean MI decreased by 28.7 (95% CI −38.5 to −18.8, *p* < 0.001) to 258.7 (SD 61.4) from the baseline. In Group B, after the fifth session of acupuncture, the MI decreased by 22.5 (95% CI −35.5 to −9.5, *p* = 0.001): from 315.9 (SD 98.5) to 293.4 (SD 90.6). After the 10th session of acupuncture, the MI decreased by 30.3 (95% CI −42.4 to −18.2, *p* < 0.001) to 285.6 (SD 94.7) from the baseline. At 1 week after completing 10 sessions of acupuncture, the mean MI decreased by 31.3 (95% CI −45 to −17.6, *p* < 0.001) to 284.6 (SD 89.2) from the baseline.

Comparing the efficacy of the first five sessions (1st to 5th) to the last five sessions (6th to 10th) in Group B, the MI decreased by 22.5 (95%CI −35.5 to −9.5) and 7.8 (95% CI −20.5 to 4.8) respectively. However, there was no statistically significant difference between these two periods (*p*-value from Scheffe's Test = 0.44).

From the linear regression analysis of the post-fifth-session treatment outcomes, apart from the different baseline MI between the groups, other factors including the duration of melasma, prior acupuncture experience, and family history of melasma did not affect the treatment outcome (see Appendix 1).

Between the two groups at 1-week follow-up post-treatment, after adjusting for the unequal baseline, there was no significant difference between the two groups (*p* = 0.67) (refer to Appendix 2).

### Melasma Area and Severity Index (MASI)

The baseline median MASI scores for the overall participants were 6 (2.6, 11.4). Group B had a higher baseline MASI score than Group A, as shown in Table 3 with the details of the MASI at each time point.

**Table 3:** Median melasma area and severity index (MASI) changes from baseline.

**MASI**	**Group A (*n* = 67)**	***p*-value**	**Group B (*n* = 39)**	***p*-value**
	**median (IQR)**		**median (IQR)**	
Baseline	5.2 (2.4, 9.0)	Reference	8.1 (4.8, 14.4)	Reference
After the 5th session	1.5 (1.2, 3.0)	<0.001^*^	4.8 (1.8, 8.0)	<0.001^*^
One week after the 5th session	1.2 (0.6, 2.1)	<0.001^*^		
After the 10th session			1.8 (1.2, 4.6)	<0.001^*^
One week after the 10th session			1.4 (1.1, 4.4)	<0.001^*^

* *p-value from Wilcoxon signed-rank test*.

*Patient-Reported Melasma-Improvement*.

In Group A, after completing five sessions of acupuncture, the median change of the MASI score was −2.9 (-5.3, −0.9) scores. Moreover, at 1 week post-fifth session, the median change of the MASI score compared with the baseline was −3.4 (−6.9, −1.2) scores. In Group B, after completing five sessions of acupuncture, the median change of the MASI score was −3.3 (−6, −0.7) scores. After completing 10 sessions of acupuncture, the median change of the MASI score compared with the baseline was −5.1 (−8.9, −2.5) scores. The median change of the MASI score between the 6th−10th sessions was −1.2 (−3, −0.6) scores. At 1 week after completing 10 sessions of acupuncture, the median change of the MASI score was −5.4 (−9.9, −3) scores.

Due to the non-normal distribution of the MASI scores, the Wilcoxon rank-sum test was used to compare the change of the MASI score between the two groups (*p* = 0.08).

The frequency distribution of patient-reported improvement scores at each time point is shown in Table 4. After the full treatment in both groups, 95.5% of Group A and 97.4% of Group B felt markedly improved or cured.

**Table 4:** Patient-reported improvement scores.

**Satisfactory score**	**Group A**	**%**	**Group B**	**%**
	**(*n* = 67)**		**(*n* = 39)**	
	**n**		**N**	
**After the 5th session**				
Worsening	-	-	-	-
No improvement	-	-	-	-
Slight improvement	-	-	2	5.7
Moderate improvement	3	4.5	4	11.4
Marked improvement	23	34.3	9	25.7
Almost or completely cured	41	61.2	20	57.1
**After the 10th session**				
Worsening			-	-
No improvement			-	-
Slight improvement			-	-
Moderate improvement			1	2.6
Marked improvement			7	18.0
Almost or completely cured			31	79.5

### Related Adverse Events From Acupuncture

There were no major adverse events. Of 106 participants, 29 (27.4%) participants reported minor side effects, wherein 14 (20.9%) participants were from group A and 15 (38.5%) participants were from group B. Five patients reported two symptoms: two had hematoma and pain, two had pain and dizziness, and the last had headache and sweating. The other 24 participants had one discomfort symptom. Group B had a risk ratio (RR) of having adverse events 1.8 times (95% CI 1–3.4, *p* =0.05) compared with group A. All the participants were willing to continue the treatment despite the adverse events.

## Discussion

Acupuncture has shown clinical benefits for melasma through MI, MASI, and patient-reported improvement. Both the MI and MASI scores of the groups showed significant improvement after five sessions of acupuncture treatment. More than 95% of all the participants subjectively reported marked improvement, which corroborates the high effective rate of acupuncture treatment for melasma from other previous studies' (13, 14).

The MASI and MI in our study were significantly reduced in both Groups A and B by 3.4 and 5.4 points, respectively for MASI, and 28.7 and 31.3, respectively for MI, at 1 week after the last session, which suggested a significant clinical magnitude of acupuncture in melasma. Recent systematic reviews and meta-analyses of oral tranexamic acid, a novel effective melasma therapy that modulates the vascular component of melasma demonstrated a decrease in MASI (1.60–1.78 points) and MI (0.69 points) (21, 22). Similarly, a meta-analysis of intense pulsed light combination therapy ranged from one to six treatment sessions in melasma reduced the MASI score only by 0.61 points (23). Furthermore, the two times per week interval of acupuncture established the shorter course of treatment compared with laser and light-based therapy. Our proposed short regimen of acupuncture is perhaps a practical and worthwhile treatment in melasma.

Since melasma is a chronic recurrent skin disease, the short practical regimen of acupuncture has many positive clinical and financial implications as in other chronic diseases. White et al. (24) mentioned the adequate dosage for acupuncture, and some meta-analysis studies proposed the adequacy criteria of at least six acupuncture sessions for low back pain (25) and chronic knee pain (26). Sangtin et al. applied a survival analysis technique in their retrospective observational data and found that 50% of stroke patients had significant improvement after eight sessions of acupuncture and reached peak improvement by 16 sessions (27). For melasma, a previous systematic review of 8 RCTs found that the number of acupuncture sessions varied from 21 to 60 over 4–16 weeks (two to seven times weekly) (19). In real practice, coming to the hospital multiple times per week for a long period is not practical both for the patients and hospital staff. In our study, the MI and MASI scores significantly decreased after 5–10 sessions with more than 95% of patients reported markedly improved or cured. Moreover, the first five sessions seem to have more magnitude of improvement than the 6th−10th sessions, although without statistically significant difference. Thus, given our setting, our protocol of 5–10 sessions is more practical than the protocol of other studies of >20 sessions (19), while still being effective with high patient satisfaction. The melasma lesion initially assessed at the first visit was more severe than at the subsequent sessions of the treatment. This might affect the outcomes that are greater in the first five sessions compared with the 6th−10th sessions. Moreover, the renewal of the epidermis takes around 28 days which is the sum of the turnover time of each compartment (28–30). Timing for the first five acupuncture sessions is approximately equal to the shedding time of the upper part of epidermis. This may result in the obvious improvement after first five sessions.

We realized that from Chinese medicine theory, one of the important basic theories is syndrome differentiation and treatment according to each syndrome. However, we found that our short fixed regimen of acupoints yielded effective results for the majority of patients, also making the treatment easier to train and perform. The treatment was likely effective because our selected acupoint included encircling and intralesional melasma area, resulting in a local increase of blood flow (31). This correlates with the evidence from the findings of an RCT that acupuncture can increase skin blood perfusion (31). Moreover, a recent systematic review of melasma treatment targeting vascular components by tranexamic acid and laser/light therapies showed promising evidence (3, 9, 11). Apart from the blood circulation and vascular component which are the recent target treatment in melasma, the latest evidence revealed that melasma is a photoaging disorder (5, 6) which is related to oxidative damage by ROS (4). Antioxidant properties of acupuncture in melasma also correlates with promising evidence for the use of antioxidants to deal with melasma in modern medicine (13, 32). Apart from the local effects, acupoints in the rest of the body acupoint—Fengchi (GB20), Hegu (LI4), Xuehai (SP10), Jusanli (ST36), Sanyinjiao (SP6), and Taichong (LR3)—have already been established to improve commonly seen syndromes of melasma patients: liver-qi stagnation, spleen-deficiency, and kidney-yin/yang-deficiency (17, 33).

In our study, the minor yet frequent adverse events of acupuncture were pain and hematoma in the facial area. Therefore, when conducting acupuncture on the facial area, clinicians should apply pressure after taking off the needles for a longer period. Higher sessions of treatment seemed to have a higher rate of adverse events.

This observational study has several limitations. The data was collected pragmatically, based on the clinical practice protocol of our acupuncture clinic. The patients would receive at least five sessions as the standard short regimen and could decide whether they were willing to continue the treatment. Hence, our study could not be generalized in terms of a direct comparison of clinical efficacies between the two groups. However, the findings from our observations could be suggestive of the dose-response relationship of the acupuncture treatment for melasma. Some factors including duration of melasma, prior acupuncture experience, and family history of melasma did not affect the clinical outcome of the treatment according to the regression result. Also, this study had a relatively short follow-up time so further research should be conducted to see how long the effects last.

## Conclusions

A short acupuncture regimen of 5–10 sessions in melasma seems to be effective and practical with minor side effects.

## Data Availability Statement

The raw data supporting the conclusions of this article will be made available by the authors, without undue reservation.

## Ethics Statement

The studies involving human participants were reviewed and approved by the Institutional Review Board (IRB), Faculty of Medicine, Chulalongkorn University. The patients/participants provided their written informed consent to participate in this study.

## Author Contributions

TS, NS, and KP conceived the idea, designed the study, collected and analyzed the data, drafted, and approved the manuscript. All authors contributed to the article and approved the submitted version.

## Funding

This study was funded by the Ratchadapiseksompotch Fund, Faculty of Medicine, Chulalongkorn University, Grant Number RA 060/60 and RA-MF-12/62.

## Conflict of Interest

The authors declare that the research was conducted in the absence of any commercial or financial relationships that could be construed as a potential conflict of interest.

## Publisher's Note

All claims expressed in this article are solely those of the authors and do not necessarily represent those of their affiliated organizations, or those of the publisher, the editors and the reviewers. Any product that may be evaluated in this article, or claim that may be made by its manufacturer, is not guaranteed or endorsed by the publisher.
