# Supplementary material for: Short Practical Regimen of Acupuncture for Melasma: A Prospective Cohort Study in a Tertiary Hospital in Thailand
Source: Front Public Health. 2021 Nov 4;9:761017. doi: 10.3389/fpubh.2021.761017 (PMC8599154; doi:10.3389/fpubh.2021.761017)
Supplement: Supplementary file 2 [file Data_Sheet_1.PDF]

STandards for Reporting Interventions in Clinical Trials of Acupuncture (STRICTA): extending the CONSORT Statement

Citation: *MacPherson H, Altman DG, Hammerschlag R, Youping L, Taixiang W, White A, Moher D; STRICTA Revision Group. Revised STandards for Reporting Interventions in Clinical Trials of Acupuncture (STRICTA): extending the CONSORT statement. PLoS Med. 2010 Jun 8;7(6):e1000261*

Table 1: STRICTA 2010 checklist of information to include when reporting interventions in a clinical trial of acupuncture (Expansion of Item 5 from CONSORT 2010 checklist)

Item	Detail	Page
**1. Acupuncture rationale**	1a) Style of acupuncture (e.g. Traditional Chinese Medicine, Japanese, Korean, Western medical, Five Element, ear acupuncture, etc)	2
	1b) Reasoning for treatment provided, based on historical context, literature sources, and/or consensus methods, with references where appropriate	2
	1c) Extent to which treatment was varied	2
**2. Details of needling**	2a) Number of needle insertions per subject per session (mean and range where relevant)	2
	2b) Names (or location if no standard name) of points used (uni/bilateral)	2
	2c) Depth of insertion, based on a specified unit of measurement, or on a particular tissue level	2
	2d) Response sought (e.g. *de qi* or muscle twitch response)	2
	2e) Needle stimulation (e.g. manual, electrical)	2
	2f) Needle retention time	2
	2g) Needle type (diameter, length, and manufacturer or material)	2
**3. Treatment regimen**	3a) Number of treatment sessions	2
	3b) Frequency and duration of treatment sessions	2
**4. Other components of treatment**	4a) Details of other interventions administered to the acupuncture group (e.g. moxibustion, cupping, herbs, exercises, lifestyle advice)	2
	4b) Setting and context of treatment, including instructions to practitioners, and information and explanations to patients	2
**5. Practitioner background**	5) Description of participating acupuncturists (qualification or professional affiliation, years in acupuncture practice, other relevant experience)	2
**6. Control or comparator interventions**	6a) Rationale for the control or comparator in the context of the research question, with sources that justify this choice	2
	6b) Precise description of the control or comparator. If sham acupuncture or any other type of acupuncture-like control is used, provide details as for Items 1 to 3 above.	2

Note: This checklist, which should be read in conjunction with the explanations of the STRICTA items provided in the main text, is designed to replace CONSORT 2010's item 5 when reporting an acupuncture trial.

STandards for Reporting Interventions in Clinical Trials of Acupuncture (STRICTA): extending the CONSORT Statement

Table 2: CONSORT 2010 checklist with the Non-pharmacological Trials Extension to CONSORT (with STRICTA 2010 extending CONSORT Item 5 for acupuncture trials)

Section/Topic	Item #	CONSORT 2010 Statement*: Checklist item[10]. Describe:	Additional items from the Non-pharmacological Trials Extension to CONSORT[14]. Add:
TITLE AND ABSTRACT			
	1.a	Identification as a randomized trial in the title	In the abstract, description of the experimental treatment, comparator, care providers, centres and blinding status.
	1.b	Structured summary of trial design, methods, results, and conclusions; for specific guidance see CONSORT for Abstracts [58,59]	
INTRODUCTION			
Background and objectives	2.a	Scientific background and explanation of rationale	
	2.b	Specific objectives or hypotheses	
METHODS			
Trial design	3.a	Description of trial design (e.g., parallel, factorial) including allocation ratio	
	3.b	Important changes to methods after trial commencement (e.g. eligibility criteria), with reasons	
Participants	4.a	Eligibility criteria for participants	When applicable, eligibility criteria for centers and those performing the interventions.
	4.b	Settings and locations where the data were collected	
**Interventions**	**5**	**The interventions for each group with sufficient details to allow replication, including how and when they were actually administered**	**Precise details of both the experimental treatment and comparator - see Table 1 for details**
Outcomes	6.a	Completely defined pre-specified primary and secondary outcome measures, including how and when they were assessed	
	6.b	Any changes to trial outcomes after the trial commenced with reasons	
Sample size	7.a	How sample size was determined	When applicable, details of whether and how the clustering by care providers or centers was addressed.
	7.b	When applicable, explanation of any interim analyses and stopping guidelines	
Randomization			
Sequence generation	8.a	Method used to generate the random allocation sequence	When applicable, how care providers were allocated to each trial group.
	8.b	Type of randomization; details of any restriction (e.g., blocking and block size)	

STandards for Reporting Interventions in Clinical Trials of Acupuncture (STRICTA): extending the CONSORT Statement

Section/Topic	Item #	CONSORT 2010 Statement*: Checklist item[10]. Describe:	Additional items from the Non-pharmacological Trials Extension to CONSORT[14]. Add:
Allocation concealment	9	Mechanism used to implement the random allocation sequence (e.g., sequentially numbered containers), describing any steps taken to conceal the sequence until interventions were assigned	
Implementation	10	Who generated the random allocation sequence, who enrolled participants, and who assigned participants to interventions	
Blinding	11.a	If done, who was blinded after assignment to interventions (e.g. participants, care providers, those assessing outcomes) and how	Whether or not those administering co-interventions were blinded to group assignment. If blinded, method of blinding and description of the similarity of interventions.
	11.b	If relevant, description of the similarity of interventions	
Statistical methods	12.a	Statistical methods used to compare groups for primary and secondary outcomes	When applicable, details of whether and how the clustering by care providers or centers was addressed.
	12.b	Methods for additional analyses, such as subgroup analyses and adjusted analyses	
RESULTS			
Participant flow (A diagram is strongly recommended)	13.a	For each group, the numbers of participants who were randomly assigned, received intended treatment, and were analyzed for the primary outcome	The number of care providers or centers performing the intervention in each group and the number of patients treated by each care provider or in each center.
	13.b	For each group, losses and exclusions after randomization, together with reasons	
Implementation of intervention			Details of the experimental treatment and comparator as they were implemented.
Recruitment	14.a	Dates defining the periods of recruitment and follow-up	
	14.b	Why the trial ended or was stopped	
Baseline data	15	A table showing baseline demographic and clinical characteristics for each group	When applicable, a description of care providers (case volume, qualification, expertise, etc.) and centers (volume) in each group.
Numbers analyzed	16	For each group, number of participants (denominator) included in each analysis and whether the analysis was by original assigned groups	
Outcomes and estimation	17.a	For each primary and secondary outcome, results for each group, and the estimated effect size and its precision (e.g., 95% confidence interval)	

**Section/Topic**	**Item #**	**CONSORT 2010 Statement*: Checklist item[10]. Describe:**	**Additional items from the Non-pharmacological Trials Extension to CONSORT[14]. Add:**
	17.b	For binary outcomes, presentation of both absolute and relative effect sizes is recommended	
Ancillary analyses	18	Results of any other analyses performed, including subgroup analyses and adjusted analyses, distinguishing pre-specified from exploratory	
Harms	19	All important harms or unintended effects in each group; for specific guidance see CONSORT for Harms [60]	
DISCUSSION			
Limitations	20	Trial limitations, addressing sources of potential bias, imprecision, and, if relevant, multiplicity of analyses	
Generalizability	21	Generalizability (external validity, applicability) of the trial findings	Generalizability (external validity) of the trial findings according to the intervention, comparators, patients and care providers and centers involved in the trial.
Interpretation	22	Interpretation consistent with results, balancing benefits and harms, and considering other relevant evidence	In addition, take into account the choice of the comparator, lack of or partial blinding, unequal expertise of care providers or centers in each group.
OTHER INFORMATION			
Registration	23	Registration number and name of trial registry	
Protocol	24	Where the full trial protocol can be accessed, if available	
Funding	25	Sources of funding and other support (e.g., supply of drugs); role of funders	

* We strongly recommend reading this Statement in conjunction with the CONSORT 2010 explanation and elaboration [11] for important clarifications on all the items. If relevant, we also recommend reading CONSORT extensions for cluster randomized trials [61], noninferiority and equivalence trials [62], herbal interventions [63], and pragmatic trials [16]. Moreover, additional extensions are forthcoming. For those and also for up-to-date references relevant to this checklist, see http://www.consort-statement.org.
